# 2544: Dietary polyunsaturated fatty acid consumption is associated with improved body composition in nonalcoholic steatohepatitis patients

**DOI:** 10.1017/cts.2017.140

**Published:** 2018-05-10

**Authors:** Hayley Billingsley, Salvatore Carbone, Justin M. Canada, Leo Buckley, Dave L. Dixon, Dinesh Kadariya, Sofanit Dessie, Benjamin W. Van Tassell, Antonio Abbate, Mohammad Siddiqui

**Affiliations:** Virginia Commonwealth University, Richmond, VA, USA

## Abstract

OBJECTIVES/SPECIFIC AIMS: Nonalcoholic steatohepatitis (NASH) is a common cause of chronic liver disease in the United States characterized by fat accumulation, inflammation, and fibrosis. Higher amounts of fat-free mass (FFM) and lower amounts of fat mass (FM) have been associated with better outcomes in several chronic diseases, recently also in NASH. Body composition is highly influenced by diet. However, the role of diet on body composition in patients with NASH is largely unknown. We hypothesized that consumption of polyunsaturated fatty acids (PUFA), healthy fatty acids mainly found in fish, nuts, and some vegetable oils, is associated with improved body composition, specifically greater FFM and lower FM, in NASH patients. METHODS/STUDY POPULATION: In total, 13 patients with histologically confirmed NASH underwent body composition testing via bioelectrical impedance analysis to estimate FFM% (% of body weight), FM% (% of body weight), and FFM/FM ratio. PUFA and saturated fat consumption was determined by standardized 5-pass 24-hour dietary recall. Correlations were computed using the Spearman rank test. RESULTS/ANTICIPATED RESULTS: Median body mass index (BMI) was 35.7 kg/m^2^ (32.8–42.7), median age of the sample was 50 years (46.3–57.3), and 73% were female. Median percent of calories from polyunsaturated fat was 6.8% (5.4–9.6). Percent of calories from PUFA was positively and significantly associated with greater FFM% (*R*=0.56, *p*=0.049), lower FM% (*R*=−0.59, *p*=0.035), and greater FFM/FM ratio (*R*=0.58, *p*=0.037). Additionally, a higher PUFA to saturated fatty acids ratio was also significantly correlated with greater FFM% (*R*=0.58, *p*=0.039), lower FM% (*R*=−0.64, *p*=0.020), and greater FFM/FM ratio (*R*=0.57, *p*=0.043). DISCUSSION/SIGNIFICANCE OF IMPACT: In patients with NASH, the consumption of PUFA is associated with higher FFM and lower FM, which suggests a protective role of these nutrients on body composition. A larger study on patients with NASH is warranted to confirm our findings on PUFA consumption and body composition, as well as to determine whether these effects will improve clinical outcomes.

